# The Beauty Health Index (BHI): A Proposed Framework for Quantifying Facial Aesthetic Health

**DOI:** 10.7759/cureus.109038

**Published:** 2026-05-17

**Authors:** Kyriaki Alevrogianni, Spero Theodorou

**Affiliations:** 1 Surgery, 401 General Military Hospital of Athens, Athens, GRC; 2 Plastic Surgery, Zucker School of Medicine at Hofstra University, New York, USA

**Keywords:** aesthetic medicine, beauty index, facial aesthetics, facial aging, skin biomarkers, skin quality

## Abstract

Facial aesthetic health is one of the most visible indicators of well-being, yet unlike cardiovascular fitness, metabolic status, or sleep quality, it lacks a standardized measurement method. Existing approaches evaluate isolated elements such as wrinkles, pigmentation, symmetry, or contour, although appearance is shaped jointly by structural anatomy, skin physiology, expression patterns, regenerative capacity, and lifestyle behaviors. No current framework integrates these components into a single interpretable score. This gap limits clinical practice: dermatologists rely on subjective grading scales, surgeons assess proportion in isolation, and consumer applications offer non-validated "beauty scores." The Beauty Health Index (BHI) is proposed as a multidomain framework for the standardized assessment and longitudinal tracking of facial aesthetic health. To develop and present a multidomain framework for the structured assessment of facial aesthetic health by integrating biologically relevant determinants into a single composite index. A structured review of literature in aesthetic surgery, dermatology, psychophysiology, regenerative biology, and lifestyle medicine informed the identification of five core domains: morphologic harmony, skin quality, expression dynamics, regenerative performance, and lifestyle influence, encompassing multiple evidence-based factors. Domain weighting was guided by literature-informed appraisal and iterative refinement to ensure balanced representation of intrinsic, extrinsic, and behavioral contributors. The BHI framework integrates 40 evidence-based subsystems across five domains into a composite score intended to reflect global facial aesthetic health. Theoretical modeling suggests proportional domain contributions and mathematically stable behavior across simulated parameter ranges, without dominance of any single subsystem. The BHI introduces a novel framework for the integrated assessment of facial aesthetic health. By combining morphologic characteristics, skin quality, expression dynamics, regenerative biology, and lifestyle factors into a unified structure, it is designed to support longitudinal evaluation, personalized planning, and structured assessment of treatment- and product-related changes. The BHI is proposed as an initial framework grounded in existing evidence, and further studies are required to evaluate its reliability, validity, and clinical applicability.

## Introduction and background

Facial appearance represents one of the most visible reflections of underlying biology and daily behavior. Although nearly every major domain of human health - cardiovascular fitness, metabolic function, sleep quality, and stress physiology - has well-defined biomarkers, facial aesthetic health still lacks an equivalent structured metric. Existing approaches evaluate isolated features, such as wrinkles, pigmentation, symmetry, or contour, even though appearance operates as a dynamic system shaped simultaneously by stable anatomic structures and rapidly modifiable variables, including lifestyle-related photoaging [1], microvascular quality [2], collagen turnover and extracellular-matrix integrity [3], hormonal rhythms influencing skin physiology [4], and expression dynamics [5]. Dermatologic science emphasizes validated skin-quality parameters such as color homogeneity [6], glycation-related dullness linked to metabolic behavior [7], periocular changes reflecting sleep disruption and stress load [8], and hair appearance as a visual modifier of perceived age and vitality [9]. Microvascular physiology further connects vascular reactivity with systemic cardiometabolic status [10]. Despite these parallel advances, no integrated and standardized quantitative framework currently exists to synthesize structural, cutaneous, regenerative, functional, and behavioral factors into a single interpretable score.

The need for such a metric is further emphasized by the absence of standardized objective assessment within the aesthetics and skincare industry, where treatment efficacy, product performance, and preventive strategies are still evaluated largely subjectively despite a global market exceeding $600 billion. Clinics and practitioners lack tools to demonstrate whether skincare, injectables, lasers, regenerative therapies, hair products, or lifestyle interventions produce measurable improvements over time, while individuals have no reliable method to track longitudinal changes in appearance.

The Beauty Health Index (BHI) is proposed as a conceptual framework to address this gap. It integrates five biologically grounded domains - morphologic harmony, skin quality, expression dynamics, regenerative performance, and lifestyle influence - into a unified, interpretable score. The BHI draws on validated research across photoaging biology [1], microvascular physiology [10], extracellular-matrix remodeling and collagen aging [3], hormonal dermatology [4], emotional-expression science [5], optical skin properties [6], metabolic contributors to dermal quality [7], and sleep-related aging mechanisms [8]. These domains are informed by established literature and reflect the multifactorial nature of facial appearance and aging.

“Beauty health” is introduced as a potential integrative domain linking facial appearance with underlying biological processes. Facial aesthetic appearance is not merely a cosmetic construct; it is a visible expression of systemic biological function. The degree to which an individual appears attractive and youthful has been associated with vascular perfusion, collagen metabolism, hormonal balance, inflammatory load, and behavioral health. Evolutionary biology has long recognized that attractiveness signals genetic fitness, immune competence, and developmental stability, while epidemiological research has demonstrated that perceived facial age predicts mortality and cardiovascular outcomes independent of chronological age. By integrating these relationships, the BHI bridges aesthetic medicine and preventive health, positioning facial appearance as a standardized and trackable indicator of overall biological wellness. This manuscript outlines the conceptual framework, component architecture, and theoretical basis of the BHI, establishing a foundation for standardized assessment and longitudinal tracking of aesthetic health.

## Review

Methods

Conceptual Approach

A structured narrative review was performed to inform the conceptual development of the BHI framework across aesthetic surgery, dermatology, psychophysiology, regenerative biology, endocrinology, sleep science, and lifestyle medicine to identify key determinants of facial aesthetic health. Candidate variables were included when supported by a mechanistic biological basis and evidence from peer-reviewed literature [11]. Additional inclusion criteria required interpretability and minimal conceptual overlap with other categories [12]. This process resulted in the identification of five core domains: morphologic harmony, skin quality, expression dynamics, regenerative performance, and lifestyle influence. Foundational evidence supporting the domain structure draws from craniofacial proportionality research [11], cutaneous biophysics [13], pigmentation and chromatic aging work [6], emotional-expression science [14], vascular physiology [10], and extrinsic aging research [15].

Subsystem Selection and Normalization

Subsystems were included only when they demonstrated strong biological grounding and reproducible measurement. For example, chromatic uniformity was incorporated due to its validated association with perceived age and its reliable quantification through colorimetric methods [6]. Pore morphology and surface microtexture were retained based on evidence demonstrating high reproducibility in digital image-based assessment [16]. Elasticity metrics were supported by studies validating the Cutometer as a reliable tool for assessing skin mechanical properties [17]. Sebum regulation was included due to standardized and validated quantification methods using sebum measurement devices [18]. Lifestyle-linked subsystems such as glycation load [7] and sleep-driven circadian variation [8] were incorporated due to strong biologic support. Subsystem scores are normalized to a 0-100 scale using literature-informed reference ranges [11].

Domain Architecture

The five domains were organized to reflect established scientific groupings. Morphologic harmony integrates proportional and structural determinants of facial balance [11]. Skin quality captures optical, mechanical, and barrier-associated traits that define cutaneous health [13] together with adjacent visual modifiers such as hair appearance [9]. Expression dynamics evaluates neuromuscular and emotional signaling patterns associated with social perception [19]. Regenerative performance encompasses microvascular and extracellular-matrix vitality linked to intrinsic repair processes [10]. Lifestyle influence quantifies modifiable behavioral factors with documented impact on skin aging and physiologic resilience [7]. This architecture enables heterogeneous biological metrics to be unified into a coherent aesthetic-health index.

Morphologic Harmony

Facial morphology represents one of the most consistently identified determinants of facial aesthetic appearance across craniofacial surgery, orthodontics, dermatology, and anthropology, with structural proportionality consistently emerging as a central component of aesthetic perception [20]. Classical anthropometric work has defined the foundational norms that guide aesthetic evaluation, including facial thirds and fifths, periorbital and nasal indices, soft-tissue relationships, and skeletal balance [21]. Subsequent research has confirmed that morphologic harmony correlates strongly with perceived attractiveness, youthfulness, and facial health across diverse populations [22].

The morphologic harmony domain integrates these validated anthropometric principles into nine structured subsystems.

The Golden Ratio Index assesses whether major facial dimensions approximate φ ≈ 1.618. Although beauty cannot depend on a single number, contemporary analyses indicate that phi-based proportions have been associated with appealing facial relationships, complementing classical neoclassical canons and conceptual templates such as the Marquardt mask [23].

Bilateral symmetry constitutes one of the most robust predictors of attractiveness, reflecting developmental stability and perceived biological fitness [24]. Quantifying asymmetry across landmarks such as endocanthion, exocanthion, alare, cheilion, zygion, gonion, and menton provides objective markers of proportional balance, with age-related soft-tissue descent and skeletal remodeling contributing to increasing asymmetry [25]. Modern 3D deviation mapping enhances the reliability of symmetry evaluation.

The Periorbital Proportion Index plays a central role in perceived youth and attentiveness. Measurements including intercanthal distance, interpupillary distance, outer canthal width, palpebral fissure height, and canthal tilt represent validated structural markers of periorbital proportion [26]. Standardized parameters, such as MRD1/MRD2 and eyelid crease morphology, support reproducible clinical assessment.

The Nasal Morphology and Projection Index incorporates anthropometric measurements, including nasal length, width, nasal index, nasofrontal angle, nasolabial angle, and columellar relationships. Nasal morphology significantly influences midfacial balance and varies across populations, highlighting the relevance of validated proportional norms [27].

The Zygomatic Projection Index evaluates bizygomatic width, malar apex position, and 3D projection patterns. Prominent malar definition correlates with youthfulness and attractiveness, while aging contributes to midface flattening and shadow-pattern alteration [28].

The Lip Morphology and Projection Index draws from proportional assessments, including upper-to-lower lip height ratios, vermillion morphology, philtral distances, and profile projection relative to aesthetic lines. Cross-cultural data confirm that lip proportion and volume significantly influence perceived attractiveness [29].

Ear-Nose Ratio Harmony reflects classical observations that ear and nose dimensions maintain proportional balance in aesthetically harmonious faces, particularly in lateral profile analysis [30].

The Jaw-Neck Definition Index incorporates mandibular width, gonial angle, ramus height, cervicomental angle, and jawline continuity. Age-related mandibular resorption, fat redistribution, and platysmal laxity progressively diminish cervicomental definition. Threshold-based CBCT data demonstrate that mandibular skeletal parameters strongly relate to age and sex, providing objective benchmarks for jawline structure [31].

To ensure cultural fairness and scientific accuracy, the BHI integrates ethnic variation across all morphologic subsystems. Facial proportions differ significantly among population groups, and reliance on Caucasian-centric norms risks systematic misclassification [30]. Incorporating validated datasets enables population-appropriate normalization of morphologic scoring, ensuring cross-cultural validity and clinical relevance.

Skin Quality

The skin quality domain of the BHI evaluates the biological state, optical behavior, and structural resilience of the skin by integrating ten validated clinical markers. These reflect both visible aging signs and functional skin-health parameters, capturing tone uniformity, radiance, elasticity, wrinkle morphology, pigmentation behavior, vascular reactivity, pore characteristics, hydration, lipid balance, and hair appearance. Because cutaneous appearance, including adjacent visual modifiers such as hair, is one of the strongest determinants of perceived age and well-being, these markers collectively provide a structured assessment of skin wellness.

Tone uniformity is a major determinant of perceived youth, with uneven chromatic distribution - such as mottling, dyschromia, or localized hyperpigmentation - strongly associated with photodamage and chronological aging. Digital color-variance analysis and spectrophotometric evaluation remain gold-standard methods for quantifying color consistency, correlating reliably with perceived age and facial attractiveness [6].

Radiance (luminance) reflects the skin’s ability to scatter and diffuse light uniformly. This parameter depends on hydration, surface microtexture, collagen arrangement, and microvascular perfusion. Studies using standardized optical imaging demonstrate that radiance can be quantified with strong reproducibility and corresponds closely with subjective perceptions of “glow” [32].

Elasticity and firmness reflect the skin’s mechanical responsiveness and resistance to deformation, governed primarily by collagen, elastin, and extracellular matrix integrity. Cutometer studies consistently validate parameters such as R2 and R7 as reliable markers of biomechanical performance [17].

Wrinkle depth is an established structural indicator of cutaneous aging. High-resolution profilometry and validated grading systems demonstrate strong reliability in quantifying wrinkle depth, density, and topographic distribution across environmental and lighting conditions [33].

Pigmentation balance assesses melanin distribution and the uniformity of light-dark patterns. Irregular pigmentation often reflects cumulative UV exposure, inflammatory history, or intrinsic melanocyte behavior. Clinical photoaging analyses highlight pigmentary heterogeneity - particularly brown spots and uneven melanin dispersion - as among the earliest and most visible markers of solar aging [34].

Redness and vascular reactivity reflect inflammation levels and superficial microvascular function. Increased erythema can indicate barrier disruption, irritation, or vascular hyperreactivity. Colorimetry and reflectance-based imaging provide accurate assessments under variable lighting, supported by standardized erythema-measurement guidelines [35]. Comparative device-validation studies further confirm the reproducibility of erythema and melanin indices across different instruments [36].

Pore density and surface microtexture influence the perception of smoothness and overall visual appeal. Enlarged pores may result from excess sebum, loss of dermal support, or photodamage. Large-scale AI-based imaging studies demonstrate that pore prevalence and severity can be quantified reliably across diverse populations and environmental conditions [37].

Hydration is a critical determinant of barrier function, radiance, and tactile softness. Aging is associated with reductions in stratum corneum water content, contributing to dullness, roughness, and fine-line severity. Established dermatologic research confirms hydration as a central pillar of visible skin quality [13].

Sebum regulation reflects lipid production and distribution, which are essential for maintaining barrier integrity, microbial defense, and surface optics. Excessive or insufficient sebum disrupts functional and aesthetic balance, and quantitative lipid measurement offers insight into overall surface physiology [38].

Hair appearance contributes meaningfully to overall facial aesthetics and influences perceptions of age, health, and attractiveness through variations in density, shaft diameter, pigmentation, and surface characteristics [9]. In addition, hair color interacts with skin tone to modulate judgments of youthfulness and vitality, demonstrating that hair functions as a visual modifier of global cutaneous appearance rather than as skin tissue itself [39].

Together, these 10 subsystems form a multidimensional evaluation of cutaneous health. By transforming visible and measurable biological traits into a unified continuous scale, the skin quality domain provides a balanced, evidence-based assessment of the skin’s optical qualities, mechanical resilience, and functional integrity within the broader BHI model.

Expression Dynamics

The expression dynamics domain of the BHI evaluates how facial musculature behaves during movement, capturing functional, emotional, and neuromuscular aspects of facial expression. Dynamic cues have been shown to influence social perception more strongly than static morphology, with studies showing that movement patterns affect judgments of vitality, authenticity, and interpersonal appeal [5]. This domain integrates seven validated subsystems: smile symmetry, eye openness, lip mobility, brow coordination, upper-lower facial synchrony, emotional authenticity, and micro-fatigue indicators.

Smile symmetry is a major determinant of perceived attractiveness and emotional well-being. Asymmetric commissure elevation may reflect neuromuscular imbalance, age-related soft-tissue changes, or reduced zygomaticus function, all of which influence social interpretations of mood and vitality [40]. Objective assessments rely on commissure-displacement ratios and left-right excursion mapping.

Eye openness reflects the dynamic function of the periorbital muscles and the eyelid aperture. Larger, more responsive aperture changes are associated with youth, alertness, and vitality, whereas narrowing, ptotic movement, or fatigued blinking correlate with aging and reduced neuromuscular performance [41]. Metrics such as blink amplitude, palpebral fissure changes, and dynamic MRD1 shifts allow standardized evaluation.

Lip mobility captures the functional range and synchronicity of the orbicularis oris and associated mimetic muscles. Reduced lip excursion may result from aging, fibrosis, volume imbalance, or previous cosmetic interventions, while excessive rigidity diminishes expressive precision [42]. Vertical and horizontal displacement mapping, along with curvature-change analysis, support objective quantification.

Brow coordination assesses how the frontalis, corrugator, and procerus activate during expressive motion. Coordinated brow elevation and contraction produce natural, believable expression profiles, whereas dissociated patterns suggest muscular fatigue, compensatory activation, or altered brow vectors [43]. Dynamic brow-arc displacement and timing-based metrics are widely used.

Upper-lower facial synchrony evaluates whether the top and bottom halves of the face move cohesively during expressions. Research in social cognition shows that synchronized segmental activation enhances perceptions of authenticity, aesthetic coherence, and emotional clarity [44]. Desynchronization may indicate fatigue, neuromuscular attenuation, or expression masking.

Emotional authenticity distinguishes genuine expressions from voluntary or posed ones. Studies using real-life emotional stimuli demonstrate that authenticity depends on coordinated activation of the zygomaticus major and periocular musculature, with specific temporal signatures differentiating genuine from posed expressions [45]. The presence or absence of periorbital involvement provides a clear physiological indicator of authenticity.

Micro-fatigue indicators represent subtle signs of neuromuscular exhaustion, reduced alertness, or transient emotional strain. These include decreased blink amplitude, slower commissure rise, delayed onset of expressional movement, and attenuated response vigor. Experimental fatigue studies validate these metrics as early dynamic markers of reduced neural and muscular efficiency [46].

Together, these seven components form a comprehensive functional assessment of expressive behavior. Because dynamic signals influence perceptions of youth, vitality, and emotional resonance, the expression dynamics domain provides essential information for the BHI, complementing structural and cutaneous metrics with real-time facial performance characteristics.

Regenerative Performance

The regenerative performance domain of the BHI evaluates deeper physiological mechanisms that sustain skin vitality and long-term tissue integrity. Unlike structural morphology or surface-level cutaneous traits, this domain evaluates microcirculatory function, collagen dynamics, and cellular renewal processes that collectively determine how effectively the skin repairs damage, maintains density, and resists aging-related decline. These biologic underpinnings are widely recognized in dermatology, regenerative biology, and aging science [2].

Vascular pattern variance assesses the organization, density, and adaptability of superficial microcirculation. Aging and chronic stress impair vasodilatory responsiveness, reduce capillary recruitment, and alter microvascular architecture, contributing to dullness, delayed healing, and compromised barrier recovery [10]. Imaging modalities such as videocapillaroscopy, laser Doppler flowmetry, and microvascular mapping demonstrate that stable, well-organized capillary networks correlate with both perceived youthfulness and biological aging markers [47].

Microcirculation efficiency reflects oxygen and nutrient delivery to the dermis and epidermis, supporting fibroblast activity, antioxidant transport, and extracellular matrix remodeling. Adequate perfusion enhances color uniformity, dermal thickness, and healing rate, while compromised perfusion is associated with reduced dermal density, slower wound closure, and inflammation-prone tissue behavior [47].

Collagen vitality measures the structural and metabolic integrity of the extracellular matrix, specifically collagen I and III architecture. Chronological aging and photoaging accelerate collagen fragmentation, reduce fiber density, and disrupt orientation patterns, leading to decreased elasticity, thinner skin, and earlier wrinkle formation [48]. Matrix degradation mediated by UV-induced metalloproteinases further compromises dermal resilience and repair capacity [49].

Cellular glow ratio reflects epidermal turnover efficiency and mitochondrial-driven energy production. Healthy epidermal renewal generates smooth surface texture, uniform reflectance and brightness, whereas delayed turnover contributes to dullness, uneven scattering and increased roughness. Mitochondrial performance regulates ATP availability for barrier repair, pigmentation homeostasis, and antioxidative defense, linking cellular energy metabolism to visible aesthetic qualities [50].

Together, these four subsystems - vascular pattern variance, microcirculation efficiency, collagen vitality, and cellular glow - form a biologically coherent model of skin regeneration. By capturing internal processes that operate beneath structural and optical features, the regenerative performance domain strengthens the BHI’s ability to evaluate both short-term aesthetic appearance and long-term facial aging trajectories.

Lifestyle Influence

The lifestyle influence domain of the BHI captures behavioral and environmental factors that measurably affect skin aging, regenerative capacity, and day-to-day facial appearance. These modifiable external elements shape inflammation levels, microvascular stability, collagen turnover, oxidative stress load, hydration regulation, and overall tissue resilience. Because lifestyle choices significantly accelerate or slow visible aging [51], this domain provides essential long-term context to the other physiologic and structural components of the BHI.

Sleep consistency is a fundamental regulator of circadian-driven DNA repair, barrier restoration, hydration rhythms, and inflammatory homeostasis. Insufficient or irregular sleep increases transepidermal water loss, decreases elasticity, impairs barrier recovery, and accelerates visible aging markers [8].

Stress and its associated cortisol fluctuations alter cutaneous microvascular behavior, increase oxidative damage, impair collagen synthesis, and slow wound healing. Chronic psychological load correlates with heightened erythema, inflammatory dysregulation, and accelerated photoaging signatures [52].

Physical activity supports cutaneous health through enhanced microcirculation, improved mitochondrial efficiency, increased antioxidant capacity, and stimulated epidermal turnover. Sedentary patterns correlate with reduced skin perfusion, slower tissue healing, decreased glow, and diminished regenerative activity [53].

Nutrition and glycation load significantly influence collagen quality. High dietary sugar promotes the formation of advanced glycation end-products (AGEs), reducing dermal elasticity and contributing to stiffness, yellowing, and early wrinkle formation [7].

Alcohol and caffeine exposure modulate hydration, vascular tone, and inflammatory behavior. Alcohol increases vasodilation, fluid loss, oxidative burden, and barrier instability, whereas caffeine can influence vascular reactivity, oxidative signaling, and stress-hormonal balance [54].

UV exposure remains the dominant extrinsic aging factor, responsible for approximately 80 percent of visible facial aging. Ultraviolet radiation breaks down collagen, increases dyspigmentation, enlarges microvascular networks, and disrupts barrier integrity [48].

Skincare routine adherence influences barrier repair, hydration balance, pigmentation uniformity, and collagen renewal. Regular use of photoprotective agents, antioxidants, humectants, and retinoids improves elasticity, reduces uneven pigmentation, and enhances epidermal thickness [13].

Treatment history captures how repeated cosmetic interventions - such as fillers, neuromodulators, lasers, and energy devices - affect long-term tissue biology. These procedures induce predictable cycles of inflammation, collagen remodeling, and microvascular adaptation, gradually modifying facial appearance and functional dynamics [49].

Smoking status is a critical determinant of skin aging, with cigarette smoke causing vasoconstriction, reduced oxygenation, impaired fibroblast function, increased matrix metalloproteinase activity, and accelerated collagen and elastin degradation. Smokers consistently show deeper wrinkles, reduced elasticity, dullness, and premature facial aging compared with non-smokers [55].

The female hormone cycle represents a predictable physiologic rhythm that influences facial appearance through cyclic changes in estrogen and progesterone. These hormonal variations may affect hydration, sebum output, microvascular perfusion, inflammatory load, and glow [40].

Together, these ten lifestyle-dependent subsystems provide a comprehensive, evidence-based framework for quantifying external influences on the BHI. They capture slow-moving but powerful determinants of long-term facial aging, offering actionable targets for intervention and prevention.

Results

The BHI demonstrates a coherent internal structure, stable mathematical behavior, and strong conceptual alignment with established biological and aesthetic principles. All 40 subsystems were selected based on predefined criteria for biological plausibility, literature support, operational clarity, and repeatability across simulated conditions. When grouped within their respective domains, the components exhibited internally consistent relationships reflecting known determinants of facial aging, cutaneous physiology, expression dynamics, and lifestyle-driven modulation.

Modeling with parameter sweeps suggests that the composite index responds proportionally to gradual changes in any single subsystem. Local improvements are associated with predictable shifts in their respective domain scores, and global BHI values change in accordance with the weighting scheme, without any single domain disproportionately dominating the outcome. This balanced behavior is consistent with established observations in skin biology, craniofacial aging, and psychophysiological research [48].

Normalization of subsystem outputs to a 0-100 scale enhances interpretability and allows changes to be visualized consistently across physiological, structural, expressive, and behavioral dimensions. Improvements in tone uniformity, hydration, or radiance are associated with comparable magnitude effects on total BHI as incremental gains in structural symmetry or cheekbone definition, reflecting how optical skin properties strongly influence perceived age and attractiveness [34].

Morphologic harmony values are consistent with established anthropometric principles, with increased proportional balance, improved zygomatic projection, or refined profile angles expected to be associated with upward shifts in the domain. Skin quality changes align with known correlations between pigmentation uniformity, radiance, hydration, and appearance-based age estimation [13]. Expression dynamics is expected to contribute modest but distinct effects, with improvements in smile symmetry, brow coordination, and upper-lower synchrony associated with potential increases in overall score, aligning with evidence that dynamic cues affect social perception and emotional clarity [44]. The regenerative performance domain is characterized by more gradual influence, consistent with biological processes governing collagen renewal, microcirculation, and mitochondrial efficiency. Changes in collagen coherence, vascular metrics, or epidermal turnover are expected to produce non-linear but directionally stable effects on overall BHI [49]. Lifestyle influence is expected to produce gradual score variation corresponding to behavioral patterns, with positive behaviors associated with upward trends and adverse exposures associated with downward trajectories, consistent with dermatologic and population-level observations [7].

These findings represent a theoretical and model-based evaluation of the BHI framework. The present work examines the internal logic, structural coherence, and expected behavior of the index under simulated conditions, rather than clinical validation in human subjects. The results should therefore be interpreted as demonstrating mathematical consistency, proportionality, and biological plausibility. Further studies will be required to evaluate the reliability, reproducibility, and clinical applicability of the BHI in real-world settings.

Discussion

This study introduces the BHI, a multidomain, biologically grounded framework designed to quantify facial aesthetic health by integrating structural, cutaneous, expressive, regenerative, and lifestyle determinants into a single interpretable score. Crucially, the BHI introduces “beauty health” as a potential integrative health-related concept, grounded in the recognition that facial attractiveness is not a superficial aesthetic construct but a visible indicator associated with underlying biological processes. How attractive and youthful an individual appears reflects the integrated status of vascular perfusion, collagen metabolism, hormonal regulation, inflammatory load, and behavioral health patterns. Unlike conventional beauty metrics or subjective grading approaches, the BHI synthesizes validated facial-anthropometry data, skin physiology markers, emotional-expression science, regenerative biology, skin and hair appearance, and behavioral health research into a standardized measurement model supported by published evidence. The BHI integrates multiple evidence-based domains into a unified framework designed to support structured assessment of facial aesthetic health. This positions aesthetic evaluation within a standardized, multidimensional approach comparable to established composite indices used in other areas of medicine, such as the Apgar score in neonatal care [56].

From a scientific and conceptual standpoint, the BHI differs from existing evaluative systems by integrating domains that are typically studied in isolation. Morphologic harmony draws from classical anthropometric work and modern proportionality research, including zygomatic projection, jaw-neck contour, orbital metrics, and profile balance, all of which influence perceived attractiveness, youthfulness, and facial symmetry. The skin quality domain incorporates validated parameters affecting perceived age, such as chromatic uniformity, radiance, elasticity, wrinkle morphology, hydration, and hair appearance, supported by optical physiology studies and dermatologic benchmarks. Expression dynamics adds a functional layer grounded in psychophysiology, recognizing that facial movement patterns, such as smile symmetry, brow coordination, and emotional authenticity, influence social judgments as strongly as static traits. Regenerative performance includes microvascular behavior, collagen metabolism, and cellular energy processes, fundamental biological systems known to drive visible skin aging and repair capacity. Lifestyle influence incorporates established external aging factors, including UV exposure, sleep patterns, glycation load, hormone cycle, and smoking, which profoundly affect long-term skin condition and aging trajectories.

Clinically, the BHI provides a comprehensive and intuitive structure for assessing baseline facial health and creating individualized treatment plans. Domain-specific scoring allows clinicians to identify whether a patient's primary concerns stem from structural imbalance, cutaneous deterioration, expressive dysfunction, regenerative decline, or lifestyle factors. For example, a patient with high morphologic harmony but low skin quality and lifestyle scores may benefit more from barrier-repair therapies, pigment correction, and behavioral interventions than from structural augmentation. Instead of relying solely on subjective clinical impressions, the BHI enables consistent documentation of progress across months or years and supports comparative evaluation between treatment modalities such as topical agents, injectables, energy-based devices, and regenerative therapies. The BHI also provides a standardized framework for monitoring treatment response over time, enabling clinicians to objectively evaluate the impact of interventions across different domains. This facilitates more precise treatment planning, supports comparison between therapeutic approaches, and enhances longitudinal patient follow-up. By translating complex multidimensional changes into a single interpretable score, the BHI allows both clinicians and patients to better understand treatment outcomes and progression.

Cultural and ethnic considerations were deliberately incorporated to prevent misclassification of normal population-specific traits as aesthetic deviations. Classical anthropometric norms - largely based on Western facial proportions - do not adequately represent global variation. The BHI integrates reference ranges from multiple ethnic groups, allowing proportionality and morphology scoring to be normalized to appropriate population baselines, enhancing fairness, accuracy, and cross-cultural validity.

The use of a composite aesthetic index raises important ethical considerations. The BHI is not intended to define individual attractiveness or personal value, but to provide a structured framework for assessing observable facial characteristics in relation to underlying biological processes. Its application must be guided by clinical judgment and contextual interpretation to avoid oversimplification or misclassification. Particular care should be taken to prevent misuse in non-clinical settings or reinforcement of unrealistic aesthetic standards. Ensuring appropriate communication, transparency, and patient-centered use is essential to minimize potential psychosocial impact.

Several limitations must be acknowledged. This initial work is conceptual and computational rather than clinical; prospective studies assessing test, retest reliability, inter-rater agreement, and correlation with expert aesthetic evaluations are needed. Some subsystems - such as emotional authenticity and cellular glow - require precise operational definitions to ensure reproducibility across imaging environments. Lifestyle factors may suffer from self-report bias; the integration of objective metrics such as actigraphy or UV dosimetry would strengthen analytic power. Furthermore, although proportionality templates like the golden ratio and phi-based masks appear in aesthetic literature, they represent broad descriptive patterns rather than universal rules and must be applied cautiously.

Future validation should include the following: intra- and inter-rater reliability testing across clinical settings, convergent evaluation with expert attractiveness ratings and apparent-age scoring, performance testing across diverse age groups and phototypes, responsiveness studies examining changes after dermatologic or surgical interventions, longitudinal observation to determine sensitivity to lifestyle modifications, and fairness and equity analyses across genders and ethnic groups. With sufficiently large datasets, machine-learning approaches, such as regularized regression or constrained modeling, may refine domain weights while preserving transparency.

Overall, the BHI offers a unified, evidence-based model that reframes aesthetic evaluation from subjective impression to structured biological analysis. By capturing proportionality, skin optics, expressive behavior, regenerative physiology, and daily habits in a single coherent metric, the BHI supports personalized care planning, objective treatment tracking, and cross-disciplinary communication. As further validation accumulates, the BHI provides a structured, evidence-based framework for aesthetic evaluation, supporting integration into clinical practice, cosmetic science, and technology-driven facial analysis.

## Conclusions

The BHI consolidates long-standing evidence from craniofacial anthropometry, skin physiology, emotional-expression science, regenerative biology, and lifestyle medicine into a single multidomain model for assessing facial aesthetic health. By reframing appearance as an integrated expression of structural anatomy, cutaneous quality, dynamic facial function, biological renewal capacity, and daily behavioral influences, the BHI provides a standardized and biologically grounded method for evaluating facial aesthetic wellness. This framework establishes a shared language for clinicians, researchers, and technology developers, enabling consistent baseline assessment, more personalized treatment planning, and structured tracking of changes over time. It supports the evaluation of interventions ranging from skincare and injectables to energy-based devices, surgical procedures, and lifestyle modifications. Its cross-disciplinary orientation connects aesthetic practice with broader concepts in aging research, regenerative medicine, and preventive health. Although this study outlines the theoretical structure, scoring architecture, and scientific rationale of the BHI, empirical validation represents the next essential step. Future research will evaluate reproducibility across settings, convergence with expert assessments, sensitivity to clinical interventions, and fairness across age groups, genders, and ethnic backgrounds. Longitudinal data will clarify how changes in skin condition, structural features, regenerative activity, expressive patterns, and lifestyle behaviors influence global BHI trajectories over time. Large, diverse datasets may also support refinement of weighting schemes and improved modeling approaches. The BHI provides a structured, evidence-based framework for integrating multiple determinants of facial aesthetic health into a unified metric, supporting personalized care planning, longitudinal tracking, and cross-disciplinary communication. By positioning facial appearance as a measurable reflection of biological and behavioral processes rather than a purely subjective construct, the BHI contributes to the development of more standardized approaches in aesthetic evaluation.
